# Introduction to the Special Issue on Eating Disorders and Obesity in Children and Adolescents

**DOI:** 10.3390/children8111065

**Published:** 2021-11-19

**Authors:** José I. Baile

**Affiliations:** Department of Psychology, Open University of Madrid (UDIMA), 28400 Madrid, Spain; joseignacio.baile@udima.es; Tel.: +34-918-561-699

In this Special Issue of *Children*, we can find several articles that present the results of various current investigations in the field of eating disorders and obesity in children and adolescents. This area requires more attention from the scientific community, due to the increased prevalence of these disorders [1], their complex clinical manifestation with great comorbidity [2] and the significant impact on the lives of people who suffer from them [3], as well as the increase in health resources that these disorders require.

The Special Issue is made up, at the moment, by ten articles, nine original articles and a review, related to various international studies, including works with European, American and Asian populations.

The etiology of these disorders is complex, so exploring different explanatory models of their different symptoms and signs is very important. This is one of the interesting objectives of the work of Sepúlveda et al. [4]: to test a model of the role of the emotional regulation of LOC eating based on a dysfunctional family environment

Similarly, it is necessary to develop new assessment instruments for these health disorders, which is why it is also one of the topics that have a place in this Special Issue. The work of Gómez-Peresmitré et al. [5] focuses on the development of the Perceived Self-Efficacy-Scale for the prevention of obesity in preteens.

Two articles, by Rabito et al. [6,7], explore the relationships between trauma experiences and eating disorders, an area that has been gaining great interest in recent years, as an explanatory source of the etiology of these disorders, since many subjects with eating disorders in adolescent and adult stages seem to have had traumatic situations early in life. Along the same lines, the work of Rojo et al. [8] analyzes the impact of stressful events in childhood on subsequent psychopathology and body weight status.

Other works included in this Special Issue analyze the unique characteristics of eating disorders in specific populations, such as athletes in the work by Martínez-Rodríguez et al. [9].

Finally, several articles, in different populations, analyze variables related to childhood obesity, eating problems or eating disorders. Variables such as social media and body esteem and their impact on adolescents are analyzed in the work of Yang et al. [10], the role of psychopathological risk factors such as body image and dissatisfaction in school-age adolescents in the work of Cabaco et al. [11], or the role of the mealtime environment and the control of food intake or feeding problems in the child development stage in the studies by Sdravou et al. [12,13].

All of the works included in this Special Issue should contribute to increasing our knowledge about the causes, manifestations, comorbidities and treatment of eating disorders and obesity in childhood and adolescence, disorders that have already received a lot of attention from the scientific community but should continue to be investigated, a goal shared by other researchers [14].

## Data Availability

Not applicable.
